# Up-regulation of tumor suppressor genes by exogenous dhC16-Cer contributes to its anti-cancer activity in primary effusion lymphoma

**DOI:** 10.18632/oncotarget.14838

**Published:** 2017-01-27

**Authors:** Yueyu Cao, Jing Qiao, Zhen Lin, Jovanny Zabaleta, Lu Dai, Zhiqiang Qin

**Affiliations:** ^1^ Department of Oncology, Research Center for Translational Medicine and Key Laboratory of Arrhythmias, East Hospital, Tongji University School of Medicine, Shanghai 200120, China; ^2^ Department of Pediatrics, Research Center for Translational Medicine and Key Laboratory of Arrhythmias, East Hospital, Tongji University School of Medicine, Shanghai 200120, China; ^3^ Department of Pathology, Tulane University Health Sciences Center, Tulane Cancer Center, New Orleans, LA 70112, USA; ^4^ Department of Pediatrics, Louisiana State University Health Sciences Center, Louisiana Cancer Research Center, New Orleans, LA 70112, USA; ^5^ Department of Genetics, Louisiana State University Health Sciences Center, Louisiana Cancer Research Center, New Orleans, LA 70112, USA

**Keywords:** KSHV, primary effusion lymphoma, tumor suppressor gene, microRNA

## Abstract

Primary effusion lymphoma (PEL) is a rare and highly aggressive B-cell malignancy with Kaposi's sarcoma-associated herpesvirus (KSHV) infection, while lack of effective therapies. Our recent data indicated that targeting the sphingolipid metabolism by either sphingosine kinase inhibitor or exogenous ceramide species induces PEL cell apoptosis and suppresses tumor progression *in vivo*. However, the underlying mechanisms for these exogenous ceramides “killing” PEL cells remain largely unknown. Based on the microarray analysis, we found that exogenous dhC16-Cer treatment affected the expression of many cellular genes with important functions within PEL cells such as regulation of cell cycle, cell survival/proliferation, and apoptosis/anti-apoptosis. Interestingly, we found that a subset of tumor suppressor genes (TSGs) was up-regulated from dhC16-Cer treated PEL cells. One of these elevated TSGs, Thrombospondin-1 (THBS1) was required for dhC16-Cer induced PEL cell cycle arrest. Moreover, dhC16-Cer up-regulation of THBS1 was through the suppression of multiple KSHV microRNAs expression. Our data demonstrate that exogenous ceramides display anti-cancer activities for PEL through regulation of both host and oncogenic virus factors.

## INTRODUCTION

Primary effusion lymphoma (PEL) is a rare B-cell malignancy that originates from B cells latently infected with Kaposi's sarcoma-associated herpesvirus (KSHV, also known as human herpesvirus-8, HHV8). PEL is almost exclusively observed in immunosuppressed patients, such as organ transplant recipients and HIV-infected patients [1]. PEL is an aggressive and incurable malignancy, with a median survival of about 6 months even under standard multi-agent chemotherapy [2]. Currently, combinational chemotherapy is the standard of care for PEL, however, the myelosuppressive effects of systemic cytotoxic chemotherapy synergize with those caused by antiretroviral therapy or immune suppression [3, 4]. Therefore, there is still an urgent need to identify new druggable targets and develop effective therapeutic strategy for those PEL patients.

Sphingolipid biosynthesis involves hydrolysis of ceramides to generate sphingosine which is subsequently phosphorylated by one of two sphingosine kinase isoforms (SphK1 or SphK2) to generate sphingosine-1-phosphate (S1P) [5, 6]. Bioactive sphingolipids, including ceramides and S1P, act as signaling molecules that regulate apoptosis and tumor cell survival [5]. In contrast to the generally pro-apoptotic function of ceramides, S1P promotes cell proliferation and survival [6]. We recently reported that one novel and selective SphK2 inhibitor, ABC294640, induces dose-dependent, caspase-mediated apoptosis in PEL cells, and suppresses PEL tumor progression *in vivo* [7]. Our additional data indicated that several exogenous ceramide and dh-ceramide species, such as C18-Cer and dhC16-Cer, also displayed anti-cancer activities for KSHV+ PEL cells *in vitro* and *in vivo* [8]. However, the underlying mechanisms for these exogenous ceramides “killing” PEL cells still require further investigation, which will be helpful to better understand PEL pathogenesis and identify more potential therapeutic targets. In the current study, we used the Illumina microarray to determine the altered gene profile in one KSHV+ PEL cell-line, BCBL-1, exposure to dhC16-Cer. We found that a subset of tumor suppressor genes (TSGs) was up-regulated from dhC16-Cer treated BCBL-1 cells. One of these elevated TSGs, Thrombospondin-1 (THBS1) was required for dhC16-Cer induced PEL cell cycle arrest. Moreover, dhC16-Cer up-regulation of THBS1 was through the suppression of multiple KSHV microRNAs expression.

## RESULTS

### Transcriptomic analysis of the gene profile altered in dhC16-Cer treated KSHV+ PEL cells

**Figure 1:**
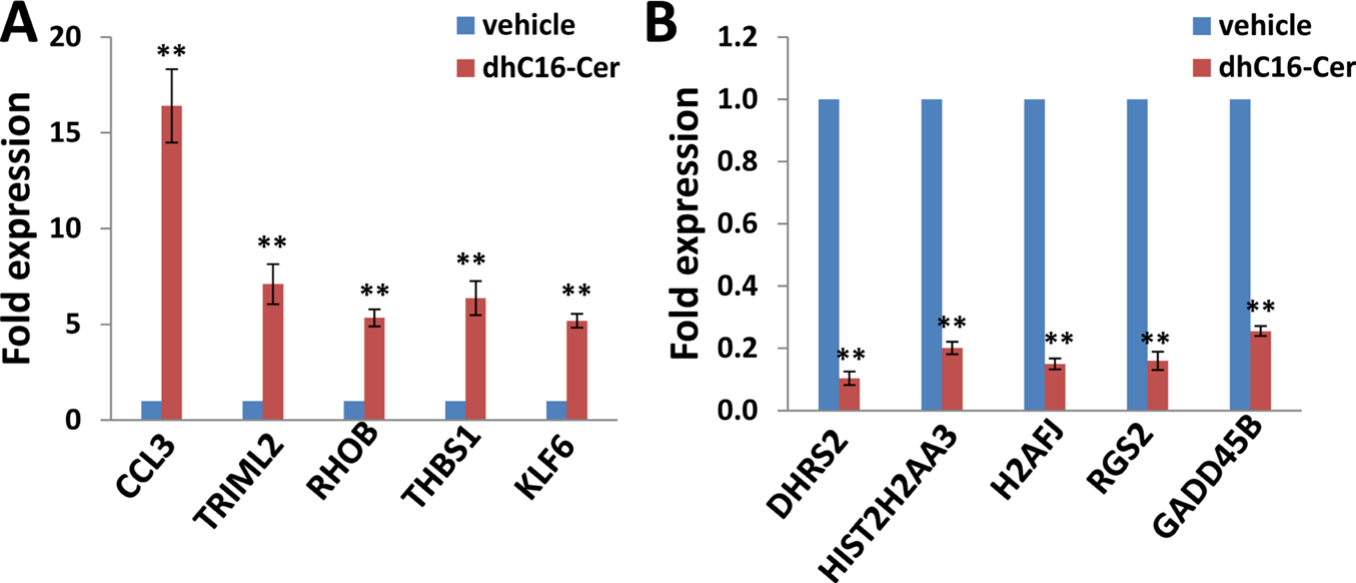
Experimental validation of gene profile alterations in dhC16-Cer treated PEL cells (**A**–**B**) The transcriptional levels of 5 selected candidate genes that were up-regulated or down-regulated in dhC16-Cer treated BCBL-1 cells from microarray data were validated by using qRT-PCR. Error bars represent the S.E.M. for 3 independent experiments. ** = *p* < 0.01 (*vs* vehicle treated cells).

**Figure 2:**
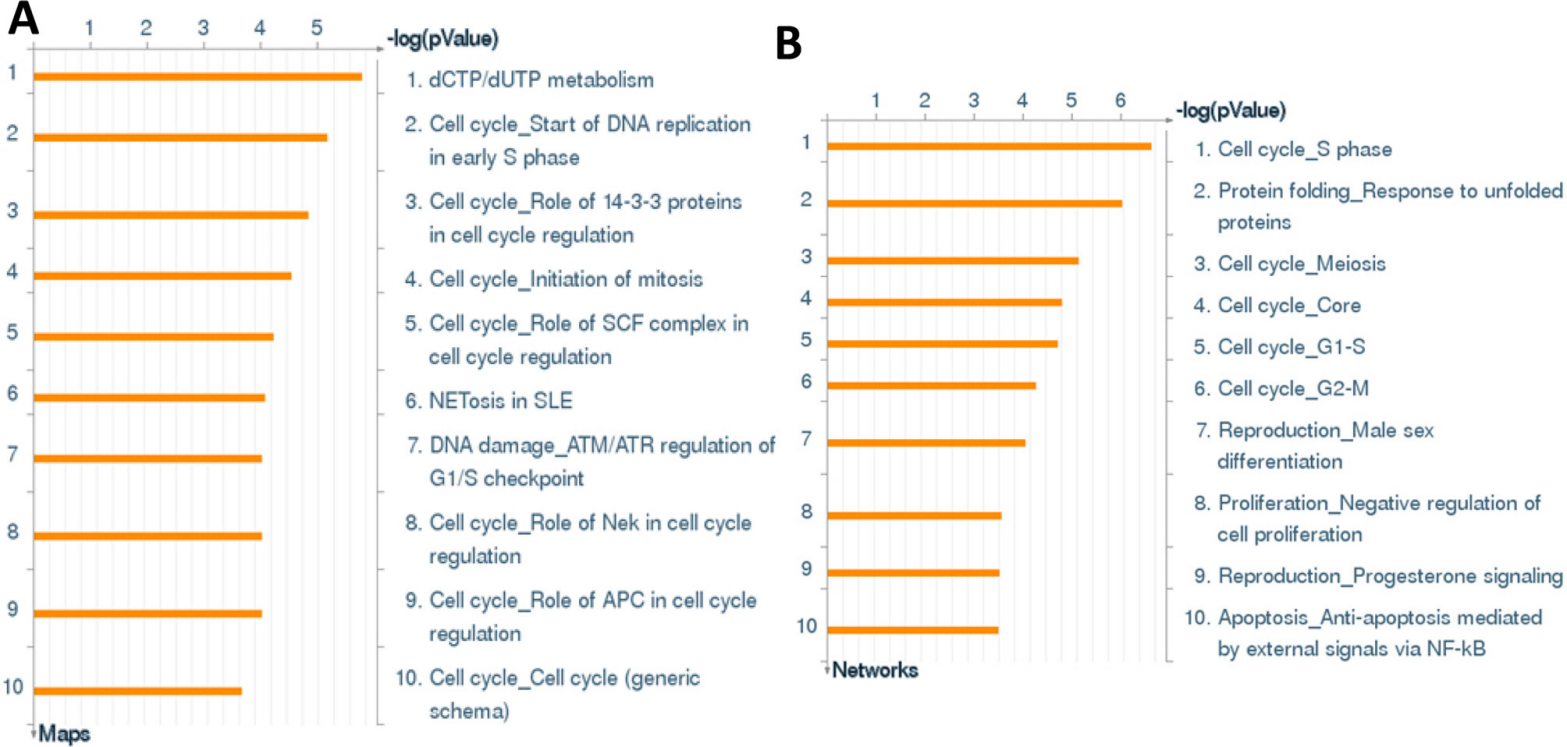
The enrichment analysis of gene profile alterations in dhC16-Cer treated PEL cells (**A**–**B**) The enrichment analysis of gene profile significantly altered (up/down ≥ 2 fold and *p* < 0.05) in dhC16-Cer treated BCBL-1 cells was performed using the Metacore Software (Thompson Reuters) Modules: Gene Ontology Processes (A) and Process Networks (B).

We first used the HumanHT-12 v4 Expression BeadChip (Illumina), which contains more than 47,000 probes derived from the NCBI RefSeq Release 38 and other sources, to study the gene profile altered within BCBL-1 cells exposure to dhC16-Cer. We found that 101 genes were significantly up-regulated and 79 were down-regulated (≥ 2 fold and *p* < 0.05) within dhC16-Cer treated BCBL-1 cells when compared to vehicle treated cells. The top 20 up-regulated or down-regulated candidate genes were listed in Tables 1 and 2, respectively. For validation of microarray analysis, we next selected 5 candidate genes from Tables 1 and 2, respectively, to perform qRT-PCR analysis. Our results indicated that all of the 10 selected genes were significantly altered in a manner comparable to those found in the microarray data, demonstrating the credibility of our results. Specifically, *CCL3*, *TRIML2*, *RHOB*, *THBS1* and *KLF6* were significantly up-regulated, while *DHRS2*, *HIST2H2AA3*, *H2AFJ*, *RGS2* and *GADD45B* were significantly down-regulated within BCBL-1 cells exposure to dhC16-Cer (Figure 1). We also performed enrichment analysis of these significantly altered candidates by using the Gene Ontology (GO) Processes and Process Networks modules from Metacore Software (Thompson Reuters). Our analysis showed that these significantly altered candidates belong to several functional categories, including cell cycle regulation, apoptosis/anti-apoptosis, cell proliferation, DNA damage and the unfolded protein response (UPR) (Figure 2). In addition, the detailed top 3 scored map (map with the lowest *p* value) based on the enrichment distribution sorted by ‘Statistically significant Maps’ set were shown in Supplementary Figures 1–3, respectively, including the dCTP/dUTP metabolism, cell cycle_start of DNA replication in early S phase and cell cycle_role of 14-3-3 proteins in cell cycle regulation.

**Table 1:** The top 20 genes up-regulated within dhC16-Cer treated KSHV+ BCBL-1 cells (*vs* vehicle-treated cells)

Gene Symbol	Gene Description	Fold
CCL3L3	Chemokine (C-C motif) ligand 3-like 3	20.96
CCL3	Chemokine (C-C motif) ligand 3	14.52
TRIML2	Tripartite motif family-like 2	6.1
RHOB	Ras homolog gene family, member B	5.19
RNF145	Ring finger protein 145	5.05
LOC731042	PREDICTED: Hypothetical protein LOC731042	4.99
LDLR	Low density lipoprotein receptor (familial hypercholesterolemia)	4.83
THBS1	Thrombospondin 1	4.21
KLF6	Kruppel-like factor 6	3.96
EIF2AK3	Eukaryotic translation initiation factor 2-alpha kinase 3	3.85
KLF2	Kruppel-like factor 2	3.72
UHRF1	Ubiquitin-like with PHD and ring finger domains 1	3.63
ZFP42	Zinc finger protein 42 homolog	3.6
APOBEC3B	Apolipoprotein B mRNA editing enzyme, catalytic polypeptide-like 3B	3.4
RRM2	Ribonucleotide reductase M2 polypeptide	3.32
LOC440871	PREDICTED: Similar to Ig kappa chain V-III region VH precursor	3.32
INSIG1	Insulin induced gene 1	3.28
DTL	Denticleless homolog	3.17
SPRR1A	Small proline-rich protein 1A	3.05
CCR7	Chemokine (C-C motif) receptor 7	3.03

**Table 2:** The top 20 genes down-regulated within dhC16-Cer treated KSHV+ BCBL-1 cells (*vs* vehicle-treated cells)

Gene Symbol	Gene Description	Fold
HSPA6	Heat shock 70kDa protein 6	0.03
HSPA7	Heat shock 70kDa protein 7	0.04
DHRS2	Dehydrogenase/reductase (SDR family) member 2	0.12
HIST2H2AA3	Histone cluster 2, H2aa3	0.16
HIST2H2AA4	Histone cluster 2, H2aa4	0.16
HSPA1B	Heat shock 70kDa protein 1B	0.18
H2AFJ	H2A histone family, member J	0.2
RGS2	Regulator of G-protein signalling 2	0.2
GADD45B	Growth arrest and DNA-damage-inducible, beta	0.21
HIST2H2AC	Histone cluster 2, H2ac	0.22
MIR1974	MicroRNA 1974	0.22
CYP4F11	Cytochrome P450, family 4, subfamily F, polypeptide 11	0.29
ACTA2	Actin, alpha 2, smooth muscle, aorta	0.29
HIST1H1C	Histone cluster 1, H1c	0.3
RAB38	RAB38, member RAS oncogene family	0.3
KLHL13	Kelch-like 13	0.32
FOSB	FBJ murine osteosarcoma viral oncogene homolog B	0.33
HIST1H4H	Histone cluster 1, H4h	0.35
SDSL	Serine dehydratase-like	0.35
LAMA5	Laminin, alpha 5	0.37

**Figure 3:**
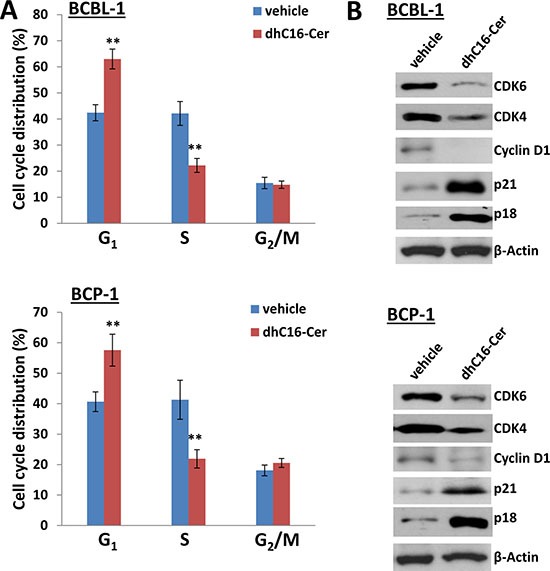
dhC16-Cer treatment causes G_1_ cell cycle arrest in PEL cells (**A**) BCBL-1 and BCP-1 were incubated with dhC16-Cer (40 μM) or vehicle for 24 h, then stained by propidium iodide (PI) and analyzed by flow cytometry. Error bars represent the S.E.M. for 3 independent experiments. ** = *p* < 0.01. (**B**) The expression of cell cycle/check-point related proteins was measured by immunoblots.

One of the major features is that many cell cycle check point or regulatory proteins were altered within dhC16-Cer treated PEL cells, implying that dhC16-Cer treatment can affect PEL cell cycle. For functional validation, we found that dhC16-Cer treatment significantly caused G_1_ cell cycle arrest as well as reducing S phase subpopulation for 2 KSHV+ PEL cell-lines, BCBL-1 and BCP-1 (Figure 3A). Immunoblots analysis confirmed that dhC16-Cer reduced the expression of check-point regulatory proteins such as CDK4, CDK6 and Cyclin D1, but increased the expression of p18 and p21 within both PEL cell-lines (Figure 3B).

### dhC16-Cer treatment up-regulates a subset of tumor suppressor genes (TSGs) from KSHV+ PEL cells

**Figure 4:**
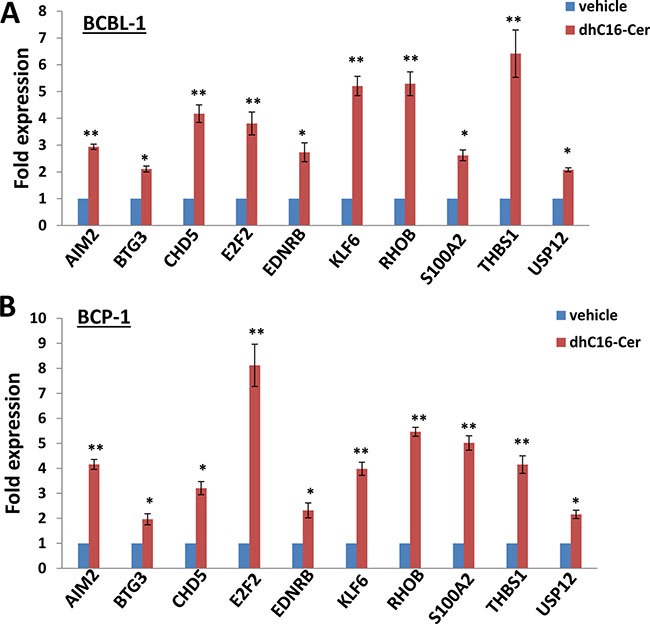
dhC16-Cer treatment up-regulates a subset of tumor suppressor genes from PEL cells (**A**–**B**) BCBL-1 and BCP-1 were incubated with dhC16-Cer (40 μM) or vehicle for 24 h, then gene transcripts were quantified by using qRT-PCR. Error bars represent the S.E.M. for 3 independent experiments. * = *p* < 0.05, ** = *p* < 0.01 (*vs* vehicle treated cells).

Interestingly, we noticed a subset of TSGs up-regulated within dhC16-Cer treated PEL cells based on our microarray data when crosslinked to the TSG database (https://bioinfo.uth.edu/TSGene/), which were listed in Supplementary Table 1. We therefore selected 10 TSGs (including *AIM2*, *BTG3*, *CHD5*, *E2F2*, *EDNRB*, *KLF6*, *RHOB*, *S100A2*, *THBS1* and *USP12*) and our qRT-PCR analysis confirmed their significant up-regulation in dhC16-Cer treated BCBL-1 and BCP-1 cell-lines, when compared to vehicle controls (Figure 4). Among these TSGs, *S100A2* and *THBS1* have been reported as direct cellular targets by multiple KSHV microRNAs [10, 11]. Repression of THBS1 expression also reduced downstream TGF-β signaling activities [10], which are related to enhance cell survival and angiogenesis for KSHV-infected cells [12, 13]. However, the cellular function of THBS1 in KSHV+ PEL cells, especially for cell cycle regulation remains unclear. So we determined to select THBS1 for further functional study.

### THBS1 is required for dhC16-Cer induced KSHV+ PEL cell cycle arrest

**Figure 5:**
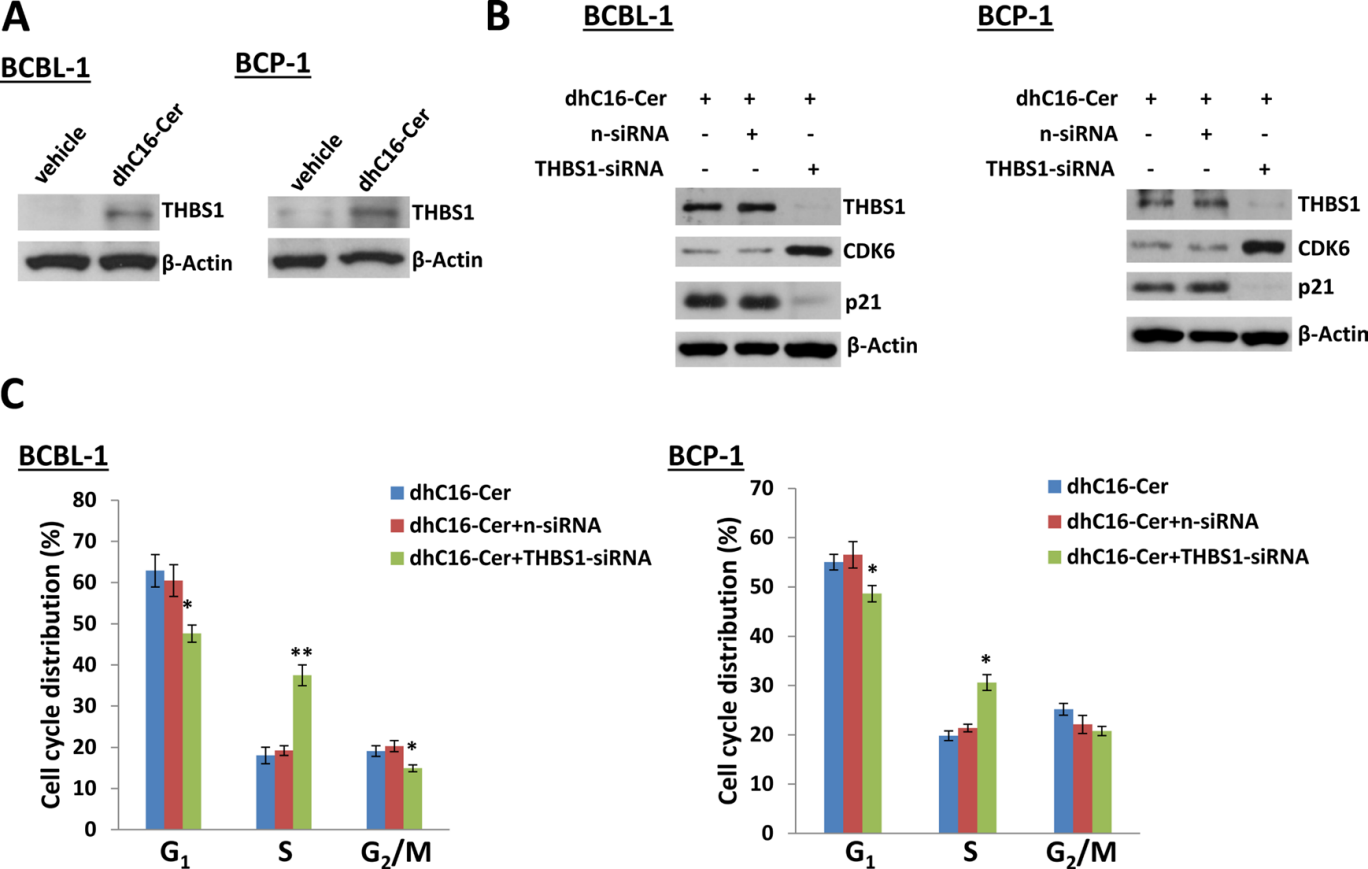
Up-regulation of THBS1 is required for dhC16-Cer caused PEL cell cycle arrest (**A**) BCBL-1 and BCP-1 were incubated with dhC16-Cer (40 μM) or vehicle for 24 h, then protein expression was measured by immunoblots. (**B**–**C**) PEL cells were first transfected with either negative control siRNA (n-siRNA) or *THBS1*-siRNA for 48 h, then incubated with dhC16-Cer (40 μM) for additional 24 h. The protein expression and cell cycle were measured by using immunoblots and flow cytometry as described above. Error bars represent the S.E.M. for 3 independent experiments. * = *p* < 0.05,** = *p* < 0.01.

We first found low basal level of THBS1 expression in both PEL cell-lines, BCBL-1 and BCP-1, as described previously [11]. In contrast to this, dhC16-Cer treatment greatly increased THBS1 expression in both PEL cell-lines (Figure 5A). We next silenced *THBS1* with specific siRNA, which simultaneously increasing CDK6 but reducing p21 expression within dhC16-Cer treated PEL cells when compared to negative siRNA transfected controls (Figure 5B). By using the flow cytometry analysis, we found that “knocked-down” *THBS1* significantly reduced G_1_ phase subpopulation while increasing S phase subpopulation for PEL cells exposure to dhC16-Cer. However, we noticed that “knock-down” *THBS1* alone could not completely rescue PEL cells from dhC16-Cer induced G_1_ cell cycle arrest, implying other cellular regulators involved in this process as well (Figure 5C).

### dhC16-Cer up-regulation of THBS1 is through repression of KSHV microRNAs

**Figure 6:**
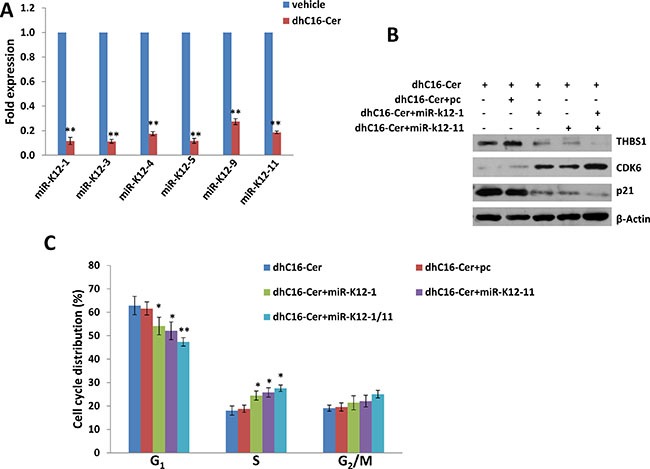
Up-regulation of THBS1 by dhC16-Cer is through suppression of KSHV microRNAs (**A**) BCBL-1 were incubated with dhC16-Cer (40 μM) or vehicle for 24 h, then KSHV microRNA transcripts were quantified using qRT-PCR as described in Methods. (**B**–**C**) BCBL-1were first transfected with control vector (pc), vectors encoding miR-K12-1 or miR-K12-11 or both for 48 h. Thereafter, cells were incubated with dhC16-Cer (40 μM) for additional 24 h. The protein expression and cell cycle were measured by using immunoblots and flow cytometry as described above. Error bars represent the S.E.M. for 3 independent experiments. * = *p* < 0.05, ** = *p* < 0.01.

KSHV encodes 25 mature microRNAs (miRNAs) derived from 12 precursor miRNAs (pre-miRs), which are highly expressed within KSHV infected cells and KSHV+ tumor tissues [14–16]. KSHV miRNAs not only promote viral latency but also modulate a lot of cellular functions important to virus infected cell survival and pathogenesis, by targeting viral genes or cellular factors [17–20]. Previous studies have reported that *THBS1* can be directly targeted by multiple KSHV miRNAs (e.g. miR-K12-1, -3, -4, -5, -6, -7, -9 and -11) at its 3′UTR region [10, 11]. Therefore, we hypothesize whether dhC16-Cer increasing THBS1 expression through regulation of KSHV miRNAs. To prove that, we first found that dhC16-Cer treatment dramatically repressed the expression of several KSHV-miRNAs, including miR-K12-1, -3, -4, -5, -9 and -11 from BCBL-1 and BCP-1 cell-lines by using qRT-PCR analysis (Figure 6A and Supplementary Figure 4). Next, we selected miR-K12-1 and miR-K12-11 for further experiments, and rescued their expression with recombinant constructs as described previously [10], which successfully reducing THBS1 and p21 expression while increasing CDK6 expression from dhC16-Cer treated BCBL-1 cells (Figure 6B and Supplementary Figure 5). By using the flow cytometry analysis, we confirmed that overexpression of miR-K12-1 and/or miR-K12-11 significantly reduced G_1_ phase subpopulation while increasing S phase subpopulation for PEL cells exposure to dhC16-Cer (Figure 6C). Together, these data demonstrated that multiple KSHV miRNAs are really involved in dhC16-Cer up-regulation of THBS1 from PEL cells and causing tumor cell cycle arrest.

## DISCUSSION

To our knowledge, this is the first transcriptomic analysis of the gene profile altered in KSHV+ PEL cell-line exposure to exogenous ceramides. We found that exogenous dhC16-Cer treatment affected many cellular genes expression, while most of them, their functional contribution to PEL pathogenesis remain largely unknown. For example, several chemokines or their receptors such as CCL3, CCL3L3 and CCR7 were up-regulated in dhC16-Cer treated PEL cells. In addition to its pro-inflammatory activities, CCL3 (also known as Macrophage inflammatory protein-1α, MIP-1α) negatively regulates the proliferation of hematopoietic stem/progenitor cells (HSPCs) [21, 22]. There is accumulating evidence supporting a crucial involvement of CCL3 in the pathophysiology of several types of leukemia arising from neoplastic transformation of HSPCs, such as chronic myeloid leukemia (CML), acute myeloid leukemia (AML) and chronic lymphocytic leukemia (CLL) [21, 23, 24]. Recent findings support CCL3 and CCL4 protein concentrations as biomarkers for B cell receptor (BCR) pathway activation and prognosis in diffuse large B cell lymphoma (DLBCL) [25]. However, the role of CCL3 and its signaling in PEL pathogenesis requires further investigation.

Notably, we found a subset of TSGs up-regulated in dhC16-Cer treated PEL cells, however, most of them have never been reported related to KSHV pathogenesis or tumorigenesis. For example, RHOB is a mainly endosomal small GTPase that regulates actin organization and vesicle trafficking, which resulting in the suppression of cancer cell proliferation, survival, invasion, and metastasis [26]. In support of its role as a negative modifier of cancer progression, targeted deletion of RHOB in mice can increase tumor formation initiated by Ras mutation [27]. Another TSG, KLF6, is frequently inactivated by loss of heterozygozity (LOH), somatic mutation, and/or decreased expression in human cancer [28]. KLF6 has been found to regulate multiple signaling pathways, including transactivation of p21 in a p53-independent manner, reduction of Cyclin D1/CDK4 complexes via interaction with Cyclin D1, inhibition of c-Jun proto-oncoprotein activities and decreased vascular endothelial growth factor (VEGF) expression [29–31].

We found that one of TSGs, THBS1 is required for dhC16-Cer induced PEL cell cycle arrest, due to suppression of KSHV miRNAs expression. Previous studies have reported that *THBS1* can be directly targeted by multiple KSHV miRNAs at its 3′UTR region [10, 11]. Since most of KSHV miRNAs promote viral latency, our recent data indicate that targeting sphingolipid metabolism by either sphingosine kinase inhibitor or exogenous ceramide species induces viral lytic gene expression from KSHV latently infected cells [8, 32]. Our previous study also found that the SphK2 inhibitor, ABC294640, repressed KSHV microRNAs expression from virus-infected endothelial cells [32]. Therefore, our findings support the possibility that sphingolipid metabolism can manipulate viral factors (e.g. miRNA) to regulate host cellular genes (e.g. TSG). The remaining question is how exogenous ceramides (e.g. dhC16-Cer) can regulate viral miRNAs synthesis/expression. Dicer is a central regulator of miRNA maturation, encoding an enzyme that cleaves double-stranded RNA or stem-loop-stem RNA into 20–25 nucleotide long small RNA [33]. One recent study has reported that Cyclin D1 (−/−) cells are defective in pre-miRNA processing which is restored by Cyclin D1 rescue [34]. Here we found that dhC16-Cer treatment caused PEL G_1_ cell cycle arrest via reducing Cyclin D1 expression. It will be interested to determine whether Cyclin D1 is involved in dhC16-Cer suppression of KSHV miRNAs expression in future study.

Our data demonstrate that exogenous ceramides display anti-cancer activities for PEL through regulation of both host and oncogenic virus factors. Targeting sphingolipid metabolism by exogenous ceramides or other ceramide analogs may represent a promising therapeutic strategy against these virus-associated malignancies.

## MATERIALS AND METHODS

### Cell culture and reagents

Body cavity-based lymphoma cells (BCBL-1, KSHV^+^/EBV^neg^) were kindly provided by Dr. Dean Kedes (University of Virginia) and maintained in RPMI 1640 medium (Gibco) with supplements as described previously [9]. BCP-1 (KSHV^+^/EBV^neg^) cells were purchased from ATCC and maintained in complete RPMI 1640 medium (ATCC) supplemented with 20% FBS. All cells were incubated at 37°C in 5% CO_2_. All experiments were carried out using cells harvested at low (< 20) passages. dhC16-Cer was purchased from Avanti Polar Lipids.

### Microarray

Microarray analysis was performed and analyzed at the Stanley S. Scott Cancer Center's Translational Genomics Core at LSUHSC. Total RNA was isolated using Qiagen RNeasy kit (Qiagen), and 500 ng of total RNA was used to synthesize dscDNA. Biotin-labeled RNA was generated using the TargetAmp-Nano Labeling Kit for Illumina Expression BeadChip (Epicentre), and hybridized to the HumanHT-12 v4 Expression BeadChip (Illumina) at 58°C for 16 h. The chip was washed, stained with streptavadin-Cy3, and scanned with the Illumina BeadStation 500 and BeadScan. Using the Illumina's GenomeStudio software, we normalized the signals using the “cubic spline algorithm” that assumes that the distribution of transcript abundance is similar in all samples. The background signal was removed using the “detection *p*-value algorithm” to remove targets with signal intensities equal or lower than that of irrelevant probes (with no known targets in the human genome but thermodynamically similar to the relevant probes). The microarray experiments were performed twice for each group and the average values were used for analysis. Common and unique sets of genes and enrichment analysis were performed using the MetaCore Software (Thompson Reuters). The microarray original data have been submitted to Gene Expression Omnibus (GEO) database (Accession number: GSE90038).

### Transfection assays

Cells were also transfected in 12-well plates using Lipofectamine 2000 (Invitrogen) for 48 h and constructs for overexpression of miR-K12-1 and miR-K12-11 as previously described [10]. Transfection efficiency was normalized through co-transfection of a lacZ reporter construct and determination of β-galactosidase activity using a commercial β-galactosidase enzyme assay system according to the manufacturer's instructions (Promega). For RNA interference, *THBS1* ON-TARGET plus SMART pool siRNA, or negative control siRNA (n-siRNA) (Dharmacon), were delivered using the DharmaFECT transfection reagent according to the manufacturer's instructions.

### Cell cycle analysis

PEL cell pellets were fixed in 70% ethanol, and incubated at 4°C overnight. Cell pellets were re-suspended in 0.5 mL of 0.05 mg/mL propidium iodide (PI) plus 0.2 mg/mL RNaseA and incubated at 37°C for 30 min. Cell cycle distribution was analyzed on a FACS Calibur 4-color flow cytometer (BD Bioscience).

### Immunoblotting

Total cell lysates (20 μg) were resolved by 10% SDS–PAGE, transferred to nitrocellulose membranes, and immunoblotted with antibodies for THBS1, Cyclin D1, CDK4, CDK6, p18, p21 (Cell Signaling) and β-Actin (Sigma) for loading controls. Immunoreactive bands were identified using an enhanced chemiluminescence reaction (Perkin-Elmer), and visualized by autoradiography.

### qRT-PCR

Total RNA was isolated using the RNeasy Mini kit (QIAGEN), and cDNA was synthesized from equivalent total RNA using a SuperScript III First-Strand Synthesis SuperMix Kit (Invitrogen) according to the manufacturer's instructions. Primers used for amplification of target genes are listed in Supplementary Table 2. Amplification was carried out using an iCycler IQ Real-Time PCR Detection System, and cycle threshold (Ct) values were tabulated in duplicate for each gene of interest in each experiment. “No template” (water) controls were used to ensure minimal background contamination. Using mean Ct values tabulated for each gene, and paired Ct values for *β-actin* as a loading control, fold changes for experimental groups relative to assigned controls were calculated using automated iQ5 2.0 software (Bio-rad). For amplification of viral miRNAs, cDNA was synthesized using the Taqman miRNA RT kit (Applied Biosystems), and qPCR was performed using the Taqman MicroRNA Assays kit (Applied Biosystems) and a 7500 Real Time PCR System. Fold changes for microRNA were calculated using paired Ct values for RNU6B as recommended by the manufacturer (Applied Biosystems).

### Statistical analysis

Significance for differences between experimental and control groups was determined using the two-tailed Student's *t*-test (Excel 8.0), and *p* values < 0.0 5 or < 0.01 were considered significant or highly significant, respectively.

## SUPPLEMENTARY MATERIALS FIGURES AND TABLES


