# Supplementary material for: Up-regulation of tumor suppressor genes by exogenous dhC16-Cer contributes to its anti-cancer activity in primary effusion lymphoma
Source: Oncotarget. 2017 Jan 27;8(9):15220–9. doi: 10.18632/oncotarget.14838 (PMC5362481; doi:10.18632/oncotarget.14838)
Supplement: Supplementary file 1 [file oncotarget-08-15220-s001.pdf]

Up-regulation of tumor suppressor genes by exogenous dhC16-Cer contributes to its anti-cancer activity in primary effusion lymphoma

Supplementary Materials

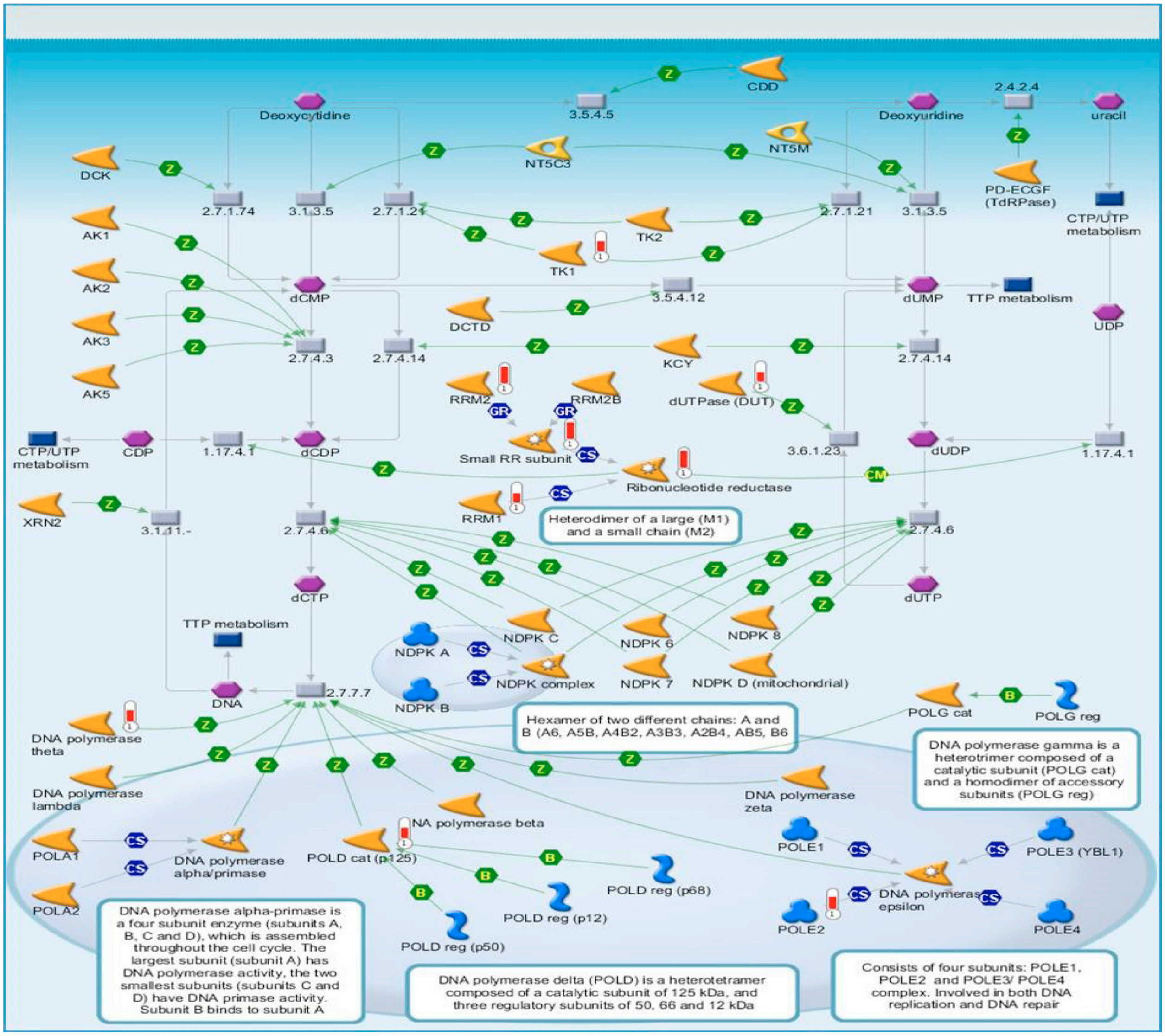

Supplementary Figure 1: The top scored maps (maps with the lowest *p* value) based on the enrichment distribution: dCTP/dUTP metabolism. Experimental data from all files is linked to and visualized on the maps as thermometer like figures. Up-ward thermometers have red color and indicate upregulated signals and down ward (blue) ones indicate downregulated expression levels of the genes. Data was produced by the MetaCore Software (Thompson Reuters).

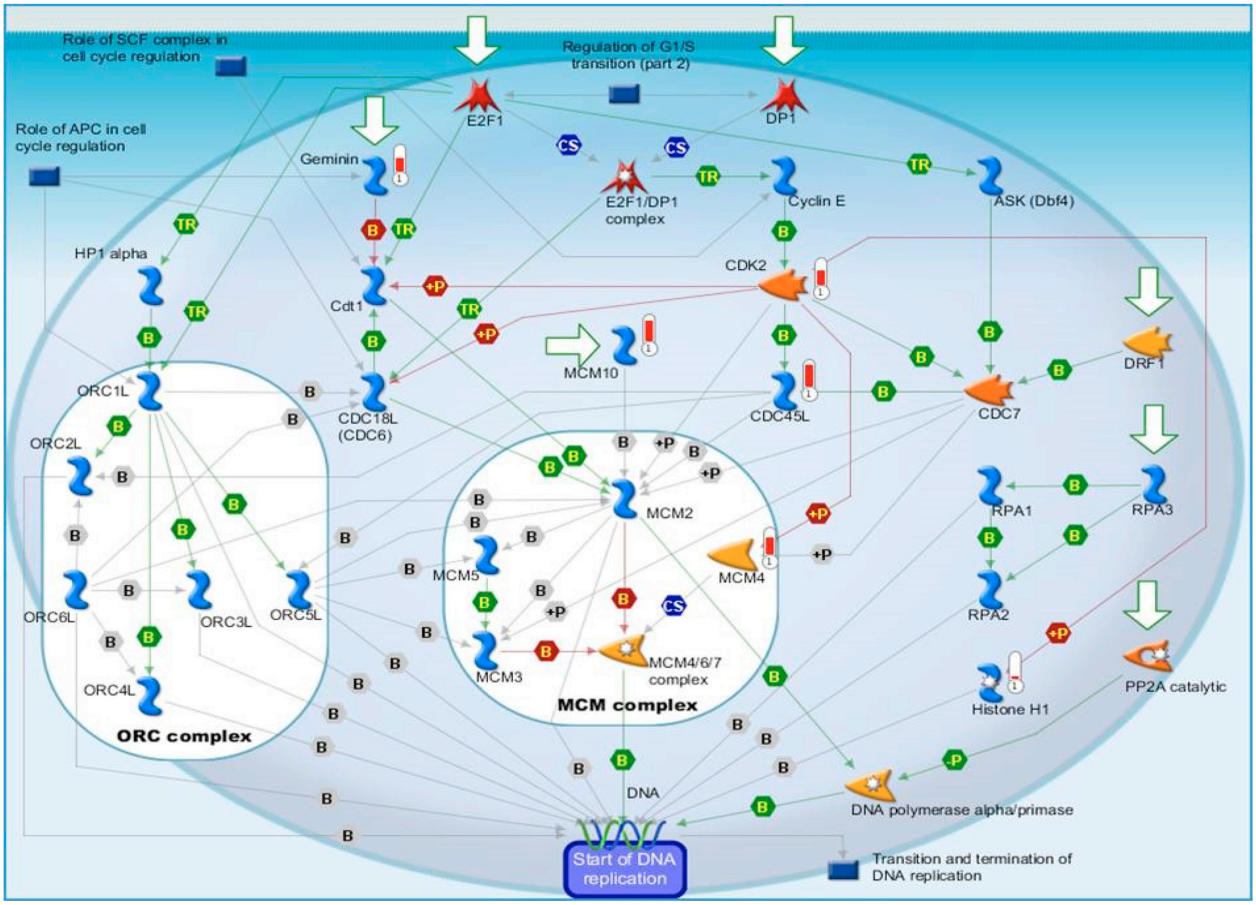

Supplementary Figure 2: The second scored maps (maps with the lowest *p* value) based on the enrichment distribution: cell cycle_start of DNA replication in early S phase. Experimental data from all files is linked to and visualized on the maps as thermometer like figures. Up-ward thermometers have red color and indicate upregulated signals and down ward (blue) ones indicate downregulated expression levels of the genes.

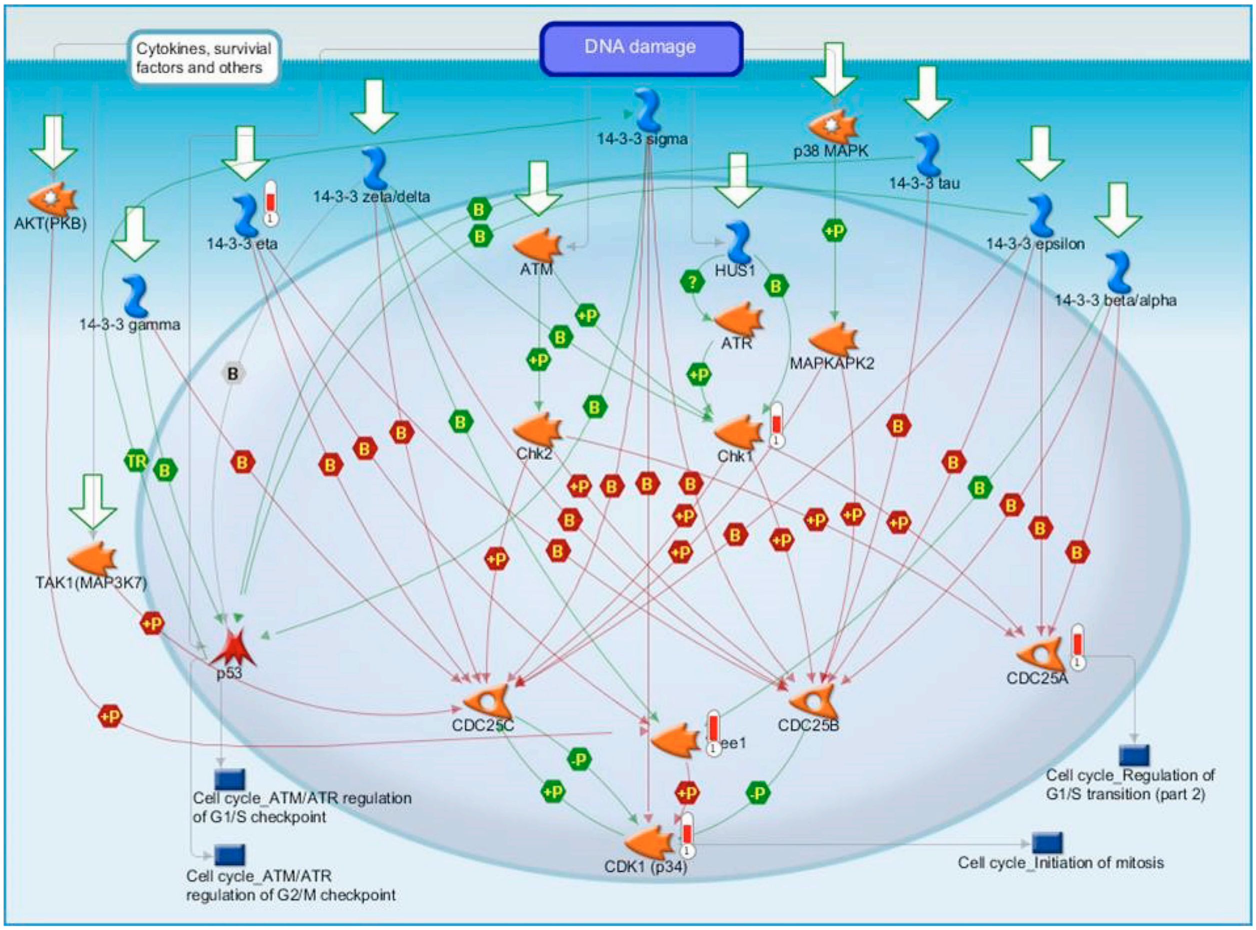

Supplementary Figure 3: The third scored maps (maps with the lowest *p* value) based on the enrichment distribution: cell cycle_role of 14-3-3 proteins in cell cycle regulation. Experimental data from all files is linked to and visualized on the maps as thermometer like figures. Up-ward thermometers have red color and indicate upregulated signals and down ward (blue) ones indicate downregulated expression levels of the genes.

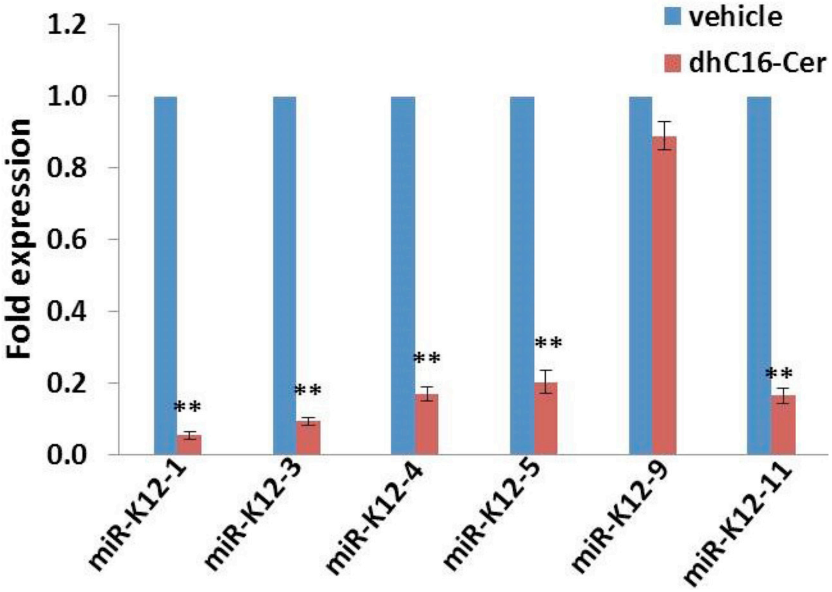

Supplementary Figure 4: Repression of KSHV microRNAs by dhC16-Cer from BCP-1 cells. BCP-1 were incubated with dhC16-Cer (40 μM) or vehicle for 24 h, then KSHV microRNA transcripts were quantified using qRT-PCR as described in Methods. Error bars represent the S.E.M. for 3 independent experiments. ** = $p < 0.01$.

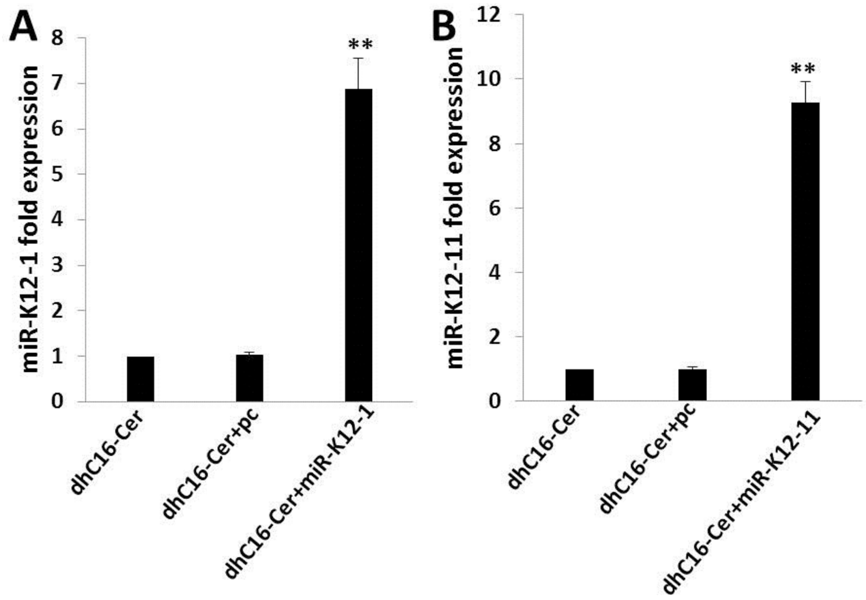

Supplementary Figure 5: Restoration of KSHV miRNA expression during dhC16-Cer treatment. BCBL-1were first transfected with control vector (pc), vectors encoding miR-K12-1 or miR-K12-11 for 48 h. Thereafter, cells were incubated with dhC16-Cer (40 μM) for additional 24 h. The KSHV microRNA transcripts were quantified using qRT-PCR as described in Methods. Error bars represent the S.E.M. for 3 independent experiments. ** = $p < 0.01$.

Supplementary Table 1: The tumor suppressor genes altered within dhC16-Cer treated KSHV+ BCBL-1 cells (*vs* vehicle-treated cells)

Gene Symbol	Gene Description
AIM2	Absent in melanoma 2
BTG3	BTG family, member 3
CADM1	Cell adhesion molecule 1
CDK2	Cyclin-dependent kinase 2
CHD5	Chromodomain helicase DNA binding protein 5
CHEK1	CHK1 checkpoint homolog
CXXC5	CXXC finger 5
E2F2	E2F transcription factor 2
EAF2	ELL associated factor 2
EDNRB	Endothelin receptor type B
EGR1	Early growth response 1
EZH2	Enhancer of zeste homolog 2
HRASLS2	HRAS-like suppressor 2
HSP90B1	Heat shock protein 90kDa beta
IGFBP4	Insulin-like growth factor binding protein 4
IKZF1	IKAROS family zinc finger 1
ITGB1	Integrin, beta 1 (fibronectin receptor, beta polypeptide)
KLF6	Kruppel-like factor 6
MSH2	MutS homolog 2, colon cancer, nonpolyposis type 1
RHOB	Ras homolog gene family, member B
S100A2	S100 calcium binding protein A2
SGMS1	Sphingomyelin synthase 1
THBS1	Thrombospondin 1
TNFRSF12A	Tumor necrosis factor receptor superfamily, member 12A
USP12	Ubiquitin specific peptidase 12

Supplementary Table 2: Primer sequences for qRT-PCR

Gene	Sequences (5′→3′)	
CCL3	*sense*	*CACTTGCTGCTGACACGC*
	antisense	*GGTCGCTGACATATTTCTGG*
TRIML2	*sense*	*CTCAGCCCTCAGTTACAGC*
	antisense	*CATCGCCATTCTTTCTTG*
RHOB	*sense*	*GCCGACATTGAGGTGGACG*
	antisense	*TGGGCACATTGGGACAGA*
THBS1	*sense*	*ACCGATTTCCGCCGATTC*
	antisense	*TGACAGTCTGGACGAGTTCTT*
KLF6	*sense*	*TCCACGCCTCCATCTTCT*
	antisense	*CATCGCCATTTCCCTTGT*
DHRS2	*sense*	*CCAAGGACATCCGGGTAA*
	antisense	*CAGAGCCGAGTGGAGTAGC*
HIST2H2AA3	*sense*	*GTGGCAAGCAAGGAGGCA*
	antisense	*CTGGAGGTGACGAGGGATG*
H2AFJ	*sense*	*CTGGCGGCGGTGTTGGAGTA*
	antisense	*CAGCACGGCCTGGATGTT*
RGS2	*sense*	*GATTGGAAGACCCGTTTG*
	antisense	*CCCTGAATGCAGCAAGAC*
GADD45B	*sense*	*CAAATCCACTTCACGCTCATC*
	antisense	*GGGACCCACTGGTTGTTGC*
AIM2	*sense*	*AAAGCCACTAAGTCAAGC*
	antisense	*TGTAGCCACTGTAGCATG*
BTG3	*sense*	*TTCACAAGGCTAGTTCGA*
	antisense	*CACAGGCTTTCAGGACAT*
CHD5	*sense*	*TCTACGACTCGGACTGGAAC*
	antisense	*GGTGGGTGAGCATCATCTT*
E2F2	*sense*	*AGACTCGGTATGACACTTCGC*
	antisense	*GCCTACCCACTGGATGTTG*
EDNRB	*sense*	*AAGGAAGTTATCTGCGAATC*
	antisense	*AGAGGGCAAAGACAAGGA*
S100A2	*sense*	*CCAAGAGGGCGACAAGTT*
	antisense	*TGATGAGTGCCAGGAAAA*
USP12	*sense*	*TTCCATAGCATAGCCACT*
	antisense	*CCATTAGGTAAACGACCA*
β-actin	*sense*	*GGAAATCGTGCGTGACATT*
	antisense	*GACTCGTCATACTCCTGCTTG*
